# A case of *Plasmodium malariae* recurrence: recrudescence or reinfection?

**DOI:** 10.1186/s12936-019-2806-y

**Published:** 2019-05-14

**Authors:** Romualdo Grande, Spinello Antinori, Luca Meroni, Michela Menegon, Carlo Severini

**Affiliations:** 1 Clinical Microbiology, Virology and Bioemergency, ASST Fatebenefratelli Sacco, Luigi Sacco Hospital, Milan, Italy; 2 Department of Biomedical and Clinical Sciences “Luigi Sacco”, University of Milan, Milan, Italy; 3 III Division of Infectious Diseases, ASST Fatebenefratelli Sacco, Luigi Sacco Hospital, Milan, Italy; 4 Department of Infectious Diseases, Istituto Superiore di Sanità, Rome, Italy

**Keywords:** *Plasmodium malariae*, Malaria, Recrudescence, Long-latency

## Abstract

**Background:**

*Plasmodium malariae* is the most neglected of the six human malaria species and it is still unknown which is the mechanism underlying the long latency of this *Plasmodium*.

**Case presentation:**

A case of PCR-confirmed *P. malariae* recurrence in a 52-year old Italian man was observed 5 months after a primary attack. In the interval between the two observed episodes of malaria the patient denied any further stay in endemic areas except for a visit to Libya, a country considered malaria-free. Genomic DNA of the *P. malariae* strain using five microsatellites (PM2, PM9, PM11, PM25, PM34) and the antigen marker of circumsporozoite (*csp*) was amplified and sequenced. Analysis of polymorphisms of the *P. malariae csp* central repeat region showed differences between the strains responsible of the first and second episode of malaria. A difference in the allele size was also observed for the sequence analysis of PM2 microsatellites.

**Conclusions:**

*Plasmodium malariae* is a challenging human malaria parasite and even with the use of molecular techniques the pathogenesis of recurrent episodes cannot be precisely explained.

**Electronic supplementary material:**

The online version of this article (10.1186/s12936-019-2806-y) contains supplementary material, which is available to authorized users.

## Background

*Plasmodium malariae* is the parasite responsible of quartan malaria with the typical periodicity of fever paroxysm observed every 72-h as detailed in a study by Camillo Golgi in 1886, but also described in the fourteenth Century by Dante Alighieri in the *Divine Comedy* (seventeen Canto of the *Inferno*) [1–3]. The parasite is widely distributed in most tropical and sub-tropical areas, with overlapping presence with *Plasmodium falciparum*, especially in sub-Saharan Africa, where it might easily be overlooked if molecular techniques such as polymerase chain reaction (PCR) are not used for diagnosis [1, 4]. Although it is well known that malaria episodes due to *P. malariae* can occur even after 30–50 years following a previous malaria attack, the mechanism responsible for its persistence and late recurrence still remains a medical mystery [5–8]. The failure to identify hypnozoites in liver biopsy of either human and animals is considered a proof that *P. malariae* is not a relapsing *Plasmodium* species, thus still giving the Bignami’s interpretation of endo-erythrocytic persistence of the parasite as the more satisfactory [3, 8–10]. However, the fact that the existence of *P. malariae* hypnozoites has never been proven is not “per se” a proof against it. For instance *Plasmodium ovale* is credited to produce hypnozoite although *P. ovale* hypnozoites have never been demonstrated biologically. Herein it is described a case of *P. malariae* infection in an Italian man occurring 5 months after a previous malaria episode despite the fact he had not travelled to a malaria-endemic region. A review of similar cases is also described together with possible explanation of this phenomenon.

## Case presentation

A 52-year-old Italian man sought care at the Emergency Department (ED) of Luigi Sacco Hospital in Milan, Italy on 14 December, 2017, complaining of a quartan pattern of fever that started 1 week before together with arthralgia and myalgia. He reported frequent trips to sub-Saharan Africa, the last one to Mozambique and several previous malaria attacks treated by himself using quinine. He reported to have not taken anti-malarial chemoprophylaxis. A chest X-ray was negative and laboratory examinations were unremarkable except for an increase of C reactive protein (50.9 mg/L) and mild thrombocytopaenia (153,000/µL). A blood smear was negative for malaria parasites as well as a rapid diagnostic test (RDT), but species-specific PCR turned positive for *P. malariae*. He was treated with a standard regimen of oral chloroquine phosphate (1 g per os initially, 500 mg 6 h after the first dose, and then 500 mg once a day on the 2nd and 3rd days of therapy). Subsequently he was in good health until the end of April when fever recurred spiking to 40 °C associated with severe headache. On 4 May, 2018 he presented to the ED of another hospital where a blood smear was positive for trophozoites of *Plasmodium* spp. He was transferred to the ED of L. Sacco hospital where a new blood smear showed scanty trophozoites of *P. malariae*; RDT was negative and species-specific PCR confirmed the diagnosis of *P. malariae*. Clinical examination was remarkable for the presence of *herpes labialis,* but otherwise negative. A chest X-ray was negative and blood examinations showed increase C-reactive protein (201 mg/L) mild anaemia (Hb 12.2 g/dL, Ht 35%), leukopaenia (WBC 3200/μL) and thrombocytopaenia (45,000/μL). In the period between the two *P. malariae* episodes he admitted only a short stay in northern Africa (Libya) without any other trip to sub-Saharan Africa. He was admitted to the Infectious Diseases Ward and treated with a 3-day course of dihydroartemisinin–piperaquine (320/40 mg) 4 tablet/day for 3 days. He was discharged on 8 May, 2018 with negative blood smear and PCR for malaria. On follow-up he had normalization of blood examinations and up to January 2019 no more recurrences of malaria.

## Methods

*Plasmodium malariae* genomic DNA was extracted from 200 μL of whole infected blood samples collected from the patient at both hospital admittances, using PureLink Genomic DNA Kit-Invitrogen. Five microsatellites (MSs) (PM2, PM9, PM11, PM25, PM34) and the antigenic marker *P. malariae* circumsporozoite (*Pmcsp*) gene were genotyped, by PCR amplification and sequencing, in *P. malariae* isolate(s) responsible for the patient’s infection in order to compare the primary infection and the second episode.

Microsatellite amplification was performed using specific primers previously described by Bruce et al. [11], adopting slight modifications in the amplification protocol (Table 1). For amplification of *Pmcsp* gene, two internal primers were designed specifically and used to amplify the central repeat region of the gene (Table 1). All PCR products were examined by gel electrophoresis and sent to Eurofins Genomics Company (Germany) for sequencing. The obtained sequences were compiled and analysed by Accelrys DS Gene Software.

**Table 1:** Primers and cycling parameters for PCR

Primers	Aminoacids	PCR cycling parameters
Pm09for	ACG ATA ATA ATA TAA ATG GGG	94 °C-30 s, 45 °C-30 s; 72 °C-1 min, 40 cycles
Pm09rev	GTT CAT AAC TTT GAT CTT AAC	
Pm11for	GGG ATA TGA ATT ACA TAC AC	
Pm11rev	CTT TAT TTG TGG TCG AGG	
Pm25for	CCA AAT AAG TGA CAT ACA AC	
Pm25rev	GAG GTA ACT TAA AAA ATT CAC	
Pm02for	GGG GCA TAA AGG AAA AAC	94 °C-30 s, 52 °C-30 s; 72 °C-1 min, 40 cycles
Pm02rev	GAA TTT TTG AAT AAC AAG AAA CC	
Pm34for	GAA TGG AAA AAT TCC TTC AG	
Pm34rev	TTG GAC AAT GAA AAA ACT AAG	
Pm MSP1for	TTC CAA AAA TTG AGG AAA TGT T	
Pm MSP1rev	TTT GGA CAA TGT CGG AAC AA	
Pm CSfor	CCC ACA AAA GCT GTT GAA AA	
Pm CSrev	TGG TGA CCA TTC CTC CGT A	

Comparison of the genetic diversity of *P. malariae* isolates collected from the patient’s two blood specimens collected on first hospital admission (14 December, 2017) and on second admission (5 May, 2018) was performed by direct sequencing of the amplified fragments of six *P. malariae* molecular markers.

A PubMed, Scopus and EMBASE literature search was performed from 1940 to 2018 with the search terms *P. malariae* AND “recrudescence” AND “recurrence” AND “relapse”. Several cases were added by cross-referencing the articles cited in the retrieved case reports. Articles in Chinese, Russian and Japanese languages were excluded.

## Results

In each of two tested DNA samples, a single amplified product was observed on agarose gel for each analysed target, suggesting the presence of a single detectable isolate for each malaria episode. The central region of *Pmcsp* gene and three MSs (PM2, PM9, PM34) were successfully sequenced in patient’s two blood specimens. The result of the sequencing of PM11 and PM25 showed the amplification of non-specific bands, resulting in a cross-reaction with human DNA, and for this reason these two molecular markers were excluded by the present analysis.

**Fig. 1:**
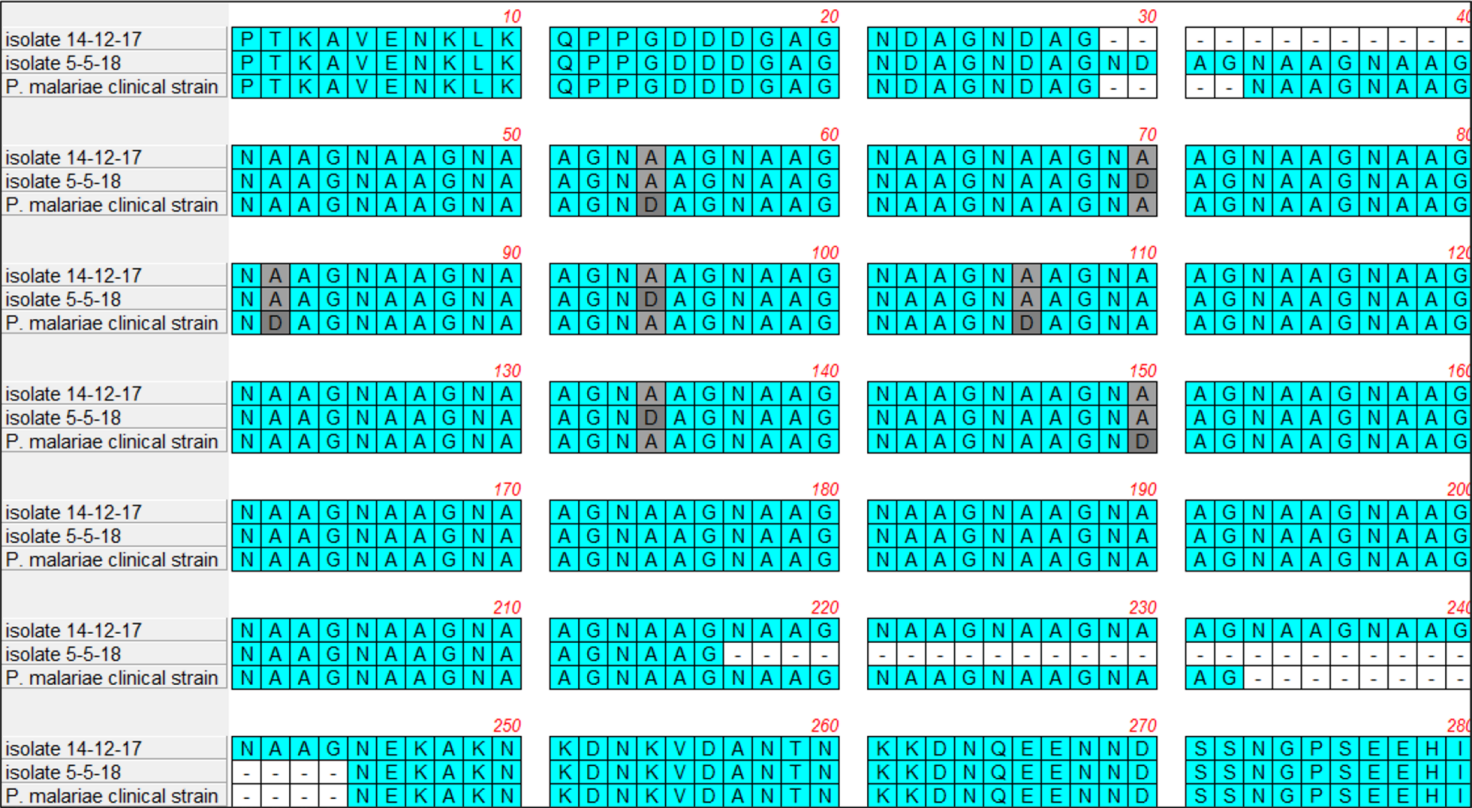
Amino acid sequence alignment of a portion of repeat region of *csp* gene of *P. malariae* isolates analyzed in the present study (Isolate 14-12-17, from first hospital admission; Isolate 05-05-18, from second hospital admission) and of a *P. malariae* isolate from an imported malaria case (*P. malariae* clinical strain)

Analysis of polymorphisms of the *Pmcsp* central repeat region resulted in the amplification of a DNA fragment of 864 base pairs (bps) (288 aminoacids) in the isolate responsible of the first episode, with a repeat region characterized by two NDAG tetrapeptide repeat units followed by 51 NAAG tetrapeptide repeat units. The isolate collected from the second episode had a more complex structure of the central repeat region (NDAG_3_ + NAAG_1_ + NAAG_5_ + NDAG_1_ + NAAG_9_ + NDAG_1_ + NAAG_20_) for a total length of 816 bps (272 aminoacids) (Fig. 1; Additional file 1: Figure S1).

**Fig. 2:**
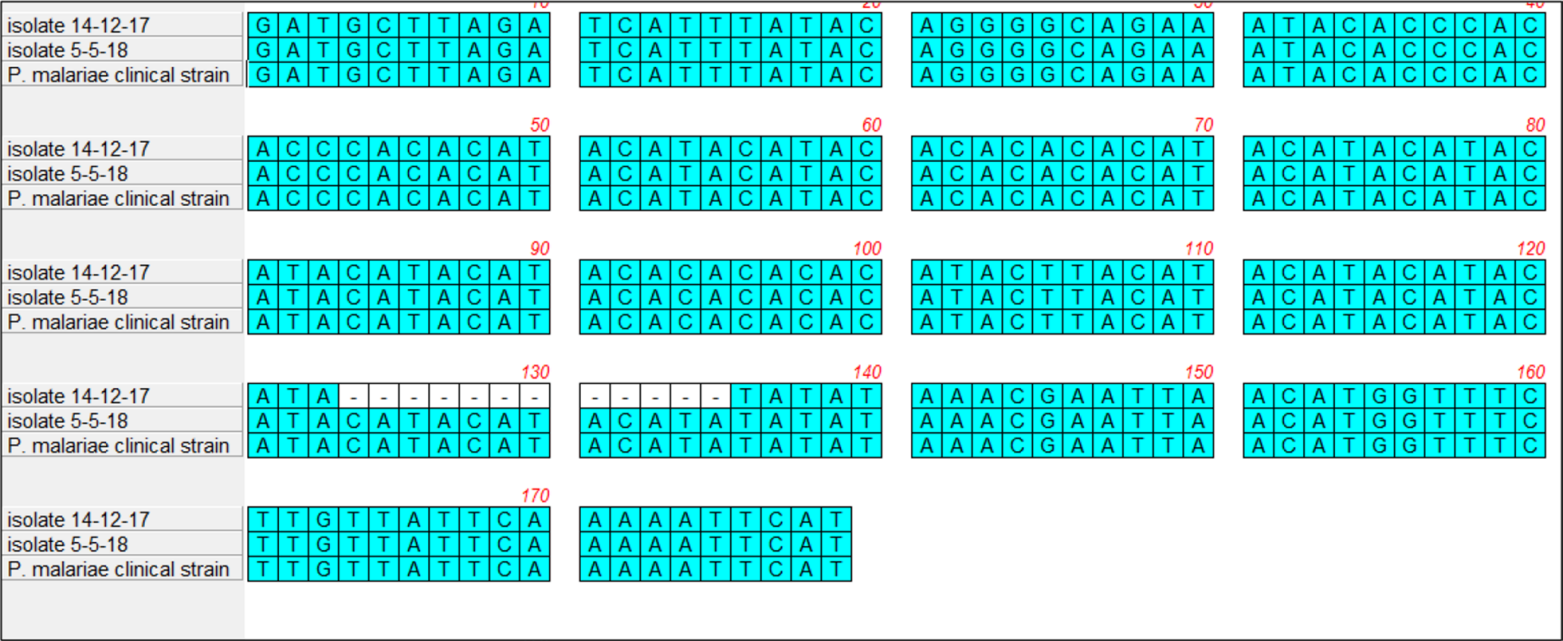
Nucleotide sequence alignment of PM2 microsatellite of *P. malariae* isolates analyzed in the present study (Isolate PM 14-12-17, from first hospital admission; Isolate PM 05-05-18, from second hospital admission) and of a *P. malariae* isolate from an imported malaria case (*P. malariae* clinical strain)

The sequence analysis of PM2 displayed two different size variants: *P. malariae* isolate collected in December’s specimen had an allele size of 192 nucleotides (nts) due to a repeat unit region of 68 nts; instead, the second isolate collected in the second hospital admission showed an allele size of 216 nts with a repeat region of 92 nts (Fig. 2: Additional file 2: Figure S2). However, both *P. malariae* isolates collected from the patient displayed the same allele for PM9 (439 nts in size) and PM34 microsatellites (295 nts).

## Review

Twenty-three case reports of *P. malariae* recrudescence were retrieved from the literature and are summarized in Table 2 [5–7, 12–31]. However, it is plausible that very ancient case reports written in languages other than English were overlooked by this research by the fact that they are not included in modern databases. The oldest retrieved report about late *P. malariae* recrudescence was published by Shute in 1944 in *The Lancet* and the same author in a review regarding imported malaria in the UK cited another two observed cases occurring after 10 and 15 years [12, 32].

**Table 2:** Case reports of *P. malariae* recrudescence

Ref/Author/year	Nationality	Age/sex	Country of Residence	Country of acquisition	Species diagnosis	Quartan fever pattern	PCR	Interval between episodes	Risk factor	Treatment
12/Shute/1944	Indian	26/F	United Kingdom	India	*P. malariae* (Microscopy)	NR	No	21 years	NR	Mepacrine
13/Spitler/1948	Italian	69/M	USA	Italy	*P. malariae* (Microscopy)	Yes	No	36 years	NR	Quinine
14/Lentini/1955	Italian	70/F	Italy	Italy	*P. malariae* (Microscopy on BM)	Yes	No	45 years	NR	Quinine
15/Creyx/1955	French	56/F	France	Macedonia	*P. malariae* (Microscopy)	Yes	No	32 years	Surgery for hydatidosis	Quinine, plasmoquine
16/Duggan and Shute/1961	English	42/M	United Kingdom	Myanmar (ex Burma)	*P. malariae* (Microscopy)	NR	No	13 years	NR	Primaquine
5/Guazzi/1963	Italian	63/F	Italy	Italy	*P. malariae* (Microscopy)	Yes	No	53 years	Splenectomy	NR
17/Tsuchida/1982	Japanese	63/M	Japan	Papua New Guinea	*P. malariae* (Microscopy)	Yes	No	36 years	Splenectomy, nephrectomy	Sulfa-pyrimethamine, primaquine
18/Hess/1993	German	46/F	Germany	Kenya	*P. malariae* (Microscopy)	Yes (2 cycles)	No	78 and 106 days	*P. falciparum* malaria	Halofantrine
6/Vinetz/1998	Greek	72/F	USA	Greece	*P. malariae* (PCR)	Yes (5 cycles)	Yes	40–70 years	Methotrexate therapy	Chloroquine
7/Chadre/2000	Trinidadian	70/M	Trinidad	Trinidad	*P. malariae* (Microscopy)	Yes (4 cycles)	No	33 years; possibly 65 years	Neurosurgery	Chloroquine + primaquine
19/Skoutelis/2000	Greek	60/F	Greece	Greece	*P. malariae* (Microscopy)	Yes	No	45 years	Treated with chlorambucil and methylprednisolone (suspected myeloproliferative syndrome)	Chloroquine
20/Morovic/2003	Serbian	61/M	Croatia (Former Yugoslavia)	Croatia	*P. malariae* (Microscopy)	Yes	No	35 years	NR	Chloroquine
21/Chim/2004	Chinese	66/F	Hong Kong	China	*P. malariae* (Microscopy)	NR	No	55 years?	Splenectomy	Chloroquine
22/Muller-Stover/2008	Nigerian	34/M	Germany	Nigeria	*P. malariae* (Microscopy; PCR)	NR	Yes	14 weeks	*P. falciparum* malaria	Atovaquone/proguanil
23/Smith/2011	Australian	59/M	Australia	Uganda	*P. malariae* (Microscopy)	NR	No	47 days	*P. falciparum* malaria	Chloroquine
24/Hedelius/2011	American	34/M	USA	Nigeria	*P. malariae* (Microscopy; PCR)	NR	Yes	14 years	NR/Concomitant diagnosis of nephrotic syndrome	Atovaquone/proguanil
25/Franken/2012	Kenyan	38/F	Germany	Kenya	*P. malariae* (Microscopy; PCR)	NR	Yes	4 months	*P. falciparum* malaria	Chloroquine
26/Hong/2012	Korean	23/F	Korea	Ghana	*P. malariae* (Microscopy; PCR)	NR	Yes	1 month	*P. malariae* malaria	First episode: chloroquine; second episode: mefloquine
27/Liang/2013	NR	20/F	USA	Uganda	*P. malariae* (Microscopy; PCR)	Yes	Yes	8 weeks	*P. malariae* malaria	First episode: chloroquine; second episode: atovaquone-proguanil
28/Kugasia/2014	Sierra Leone	65/M	USA	Sierra Leone	*P. malariae* (initially misidentified as *P. falciparum*)	No	No	25 months	*P. malariae* malaria	First episode: quinine + clindamycin Second episode: chloroquine
29/Visser/2016	Dutch	NR/M	The Netherlands	Uganda	*P. malariae* (Microscopy; PCR)	No	No^a^	2 months	*P. malariae* malaria	Chloroquine
30/Rutledge/2017	Ugandan	31/M	Australia	Uganda	*P. malariae* (Microscopy; PCR)	No	Yes	52 days	*P. malariae* malaria	First episode: artemether/lumefantrine Second episode: hydroxychloroquine + primaquine
31/Islam/2018	Ivory Coast	23 months/M	USA	Liberia	*P. malariae* (Microscopy; PCR)	No	Yes	4 months	*P. malariae* malaria	First episode: Chloroquine Second episode: chloroquine Third episode: atovaquone–proguanil

*M* male, *F* female, *PCR* polymerase chain reaction, *NR* not reported, *BM* bone marrow

^a^PCR was not employed for the recurrence diagnosis

In the oldest cases recorded the diagnosis of *P. malariae* infection was based on microscopy only and the long latency before the recrudescence was inferred by the notion about elimination of malaria in the country of origin or by the last time in which the patients lived in or travelled to an endemic area [5, 6, 12–17, 19–21]. No difference of gender was observed in the cases reported in the literature and the median age of patients was 59 years. A quartan fever paroxysm was reported in 73.3% of described cases for whom this information was available (11/15) [6, 7, 13–15, 17–19, 26]. Malaria was acquired in Europe (before its elimination) in 7 cases, in sub-Saharan Africa in 11 cases, in Southeast Asia in 3 cases, and in Trinidad in the remaining case. The attack of malaria was precipitated by surgery in five patients (splenectomy in 3 cases), and immunosuppressive therapy in 2 patients [5–7, 15, 17, 19, 21]. An episode of falciparum malaria preceded the recrudescence by *P. malariae* in 4 patients consistent with the possibility of initially overlooked mixed infections [18, 22, 23, 25]. Six patients had a documented episode of malariae malaria 4 weeks to 25 months before the diagnosis of recrudescence [26–31]. Splenomegaly was documented in 9 patients (36.4%) and in two cases splenectomy was undertaken as diagnostic evaluation [5, 6, 14, 18–21, 31]. At the time of *P. malariae* recrudescence diagnosis, a nephritic syndrome was observed in two patients [21, 24]. Chloroquine was the drug most frequently employed for treatment (12 patients, 52.2%) followed by quinine (4 patients) [6, 7, 13–15, 19–21, 23, 25–31].

## Discussion

*Plasmodium malariae* is the most neglected among *Plasmodium* species responsible of human malaria, being frequently undetected due to the very low parasitaemia it causes. Only recently a draft nuclear genome and a high-quality reference genome of *P. malariae* have been made available but nevertheless this *Plasmodium* remains the most mysterious among human malaria parasites and its long-term persistence has so far been elusive to any convincing explanation [33, 34]. Moreover, the existence among African apes and New World monkeys of two very similar species based on morphologic characteristics (i.e., *Plasmodium rhodaini* and *Plasmodium brasilianum*) has raised the question whether *P. malariae* should be considered a zoonosis, an anthroponosis or as inferred by Lalremruata et al., at least in South America, *P. malariae* and *P. brasilianum* are a single anthropozoonotic species [35–37].

Recurrences of *P. malariae* infection even after more than 50 years of latency have been described in old malaria literature (Table 2) but the availability of molecular biology techniques nowadays allowed a better classification of such episodes as a consequence of recrudescence or re-infection [5–7, 12–31].

Herein it is described a case of an Italian man who presented two symptomatic PCR-confirmed episodes of *P. malariae* infection occurring 5 months apart. His medical history was notable for several previous episodes of malaria acquired during his frequent travels to sub-Saharan Africa, usually with self-treatment with quinine. In the interval between the two observed episodes of malaria the patient denied any further stay in endemic areas except for a visit to Libya, a country considered malaria-free, thus giving the hypothesis of a recrudescence as the most plausible. Interestingly, genotyping of four *P. malariae* specific genetic markers indicated substantial genetic diversity of the two *P. malariae* strains responsible for the first and the second malaria episode. In this context the possibility of a de-novo infection acquired in Libya cannot be discarded and the recent description in Italy by Martelli et al. [38] of two Malian immigrants diagnosed with falciparum malaria in Italy after a long stay (3 to 5 years) in Libya might be concordant with this hypothesis.

Alternatively, in the case of recrudescence, one can consider the possibility of chloroquine-resistance or inadequate drug concentration. To the best of knowledge clinical chloroquine-resistant *P. malariae* has been reported only in a study conducted in Indonesia and in a single anecdotal case acquired in Africa [28, 39]. In the former study one patient had persistent parasitaemia on day 28 and two had persistent parasitaemia on day 8. However, Collins and Jeffery retrospectively analysed the curve of clearance of *P. malariae* among neurosyphilis patients infected with *P. malariae* and subsequently treated with chloroquine and showed that an extended clearance time is frequent with this species, concluding that is not indicative of resistance to chloroquine [40].

However, recurrence of *P. malariae* in cases of mixed malaria infections in which *P. malariae* was initially overlooked and the patients were treated for *P. falciparum* infection with mefloquine, halofantrine, quinine, or artemether/lumefantrine (AL) have been previously reported [18, 22, 23, 28]. More recently, Rutledge et al. [30] described a late recrudescence of *P. malariae* in which the minority initial subpopulation survived after treatment with AL and they postulated the need to use artemisinin combination therapy (ACT) with long half-life partner drug and longer follow-up of patients with *P. malariae*. Another possibility to be investigated as proposed for *P. falciparum* is the sequential use of two artemisinin-based combination regimens [41]. As far as the described patient, although the malaria infection was acquired in Africa where a single possible *P. malariae* chloroquine-resistant episode has been described, the decision was to treat the second episode of malaria with dihydroartemisinin–piperaquine (DHP) with no malaria recurrence after 8 months of follow-up [26]. However, given the long latency described for *P. malariae* infections it is presently unknown for how long a patient should be followed and, in clinical practice, among travellers with imported malaria follow-up longer than 4 weeks seems unfeasible [5–7, 12–17, 19–21, 23]. More recently, in a study aimed to investigate the biological basis of breakthrough *P. malariae* among travellers using atovaquone–proguanil chemoprophylaxis, Teo and co-workers showed the absence of mutation in the parasite locus *pmcytb* associated with recrudescence of *P. falciparum* [42]. These investigators hypothesized again the possibility that *P. malariae* is a relapsing parasite with the capacity to undergo latent hypnozoites in the liver responsible of new erythrocytic sexual replication years after the initial infection. Based on the life span of erythrocytes (110 days) and of hepatocytes (150–300 days), Richter et al. [43] concurred with the hypothesis of the liver as the possible reservoir of *P. malariae*. However, Garnham, based on several observations concluded that “*P. malariae* are recrudescences of persisting blood forms of the respective parasites” [3, 8, 44].

Moreover, it is hard to explain how a dormant liver parasite is responsible of malaria transmitted by blood transfusion by an asymptomatic blood donor as long as 44 years after the last exposure in a malaria endemic area [45–47]. The interesting report by Rutledge and co-workers who showed that the recrudescent isolate in the patient they described was a single clone present at low density in the initial *P. malariae* infection, raised also the possibility that this patient harboured several different population of *P. malariae* acquired at different time frames with different fitness to anti-malarial drugs, one of which was capable of emerging when the susceptible ones were eliminated [30].

The review of the literature confirms the importance of immunity in controlling long-term latency of *P. malariae* with a state of balanced parasitism which can be abruptly broken by stress events, such as surgery or removing the spleen or by treatment with immunosuppressive drugs, each one responsible for recrudescence of malaria [5–7, 15, 17, 19, 21]. However, the precipitating event in several other cases remain unexplained [12–14, 16, 18, 20, 22–31]. Also intriguing is the ability of *P. malariae* to escape drugs which are blood schizontocidal for *P. falciparum* despite the very low parasitaemia that the former parasite produces. It can be speculated that in view of the long pre-patent period of *P. malariae* the duration of therapy against this *Plasmodium* should be increased and when using an artemisinin combination, as suggested by Rutledge et al., a partner drug that is slowly eliminated should be preferred [30, 33].

## Conclusion

It is described a case of probable *P. malariae* recrudescence, together with explaining hypothesis and a review of the literature of long-latency recrudescences. One-hundred and sixty-five years after its description *P. malariae* remains the most mysterious of the human malaria parasites and as suggested by Shute “it provides an almost perfect example of successful parasitism, without the frequent change of host need by other species of human malaria” [12]. Several recent thorough reviews about *P. malariae* persistent parasitism take again the hypnozoite hypothesis based on the fact that the “not proven existence” (as for *P. ovale*) should not be considered as proof for the non-existence of *P. malariae* hypnozoites and the need to search for an alternative place of dormancy, such as the spleen [25, 43, 48–50].

## Additional files

**Additional file 1: Figure S1.** Phylogenetic tree inferred from CSP nucleotide sequence alignment obtained from *P. malariae* isolates analyzed in the present study (Isolate PM 14-12-17; Isolate PM 05-05-17) and from 12 representative CSP gene sequences retrieved from the GenBank database. *P. vivax* CSP gene sequence (Genbank accession number AJ295636) was also included in the analysis and used as an outgroup. Phylogenetic analysis was done using the neighbour-joining method constructed using the neighbor-joining method by bootstrapping with 1000 replicates, and phylogenetic distances were measured by Tajima-Nei model, using the Accelrys DS Gene software package (Accelrys Inc., San Diego, CA, USA).

**Additional file 2: Figure S2.** Phylogenetic tree inferred from PM2 microsatellite sequence alignment obtained from *P. malariae* isolates analyzed in the present study (Isolate PM 14-12-17; Isolate PM 05-05-17) and from all representative sequences of PM2 microsatellite retrieved from the GenBank database. Phylogenetic analysis was done using the neighbour-joining method constructed using the neighbor-joining method by bootstrapping with 1000 replicates, and phylogenetic distances were measured by Tajima-Nei model, using the Accelrys DS Gene software package (Accelrys Inc., San Diego,CA,USA). Reference: Tajima F, Nei M. Estimation of evolutionary distance between nucleotide sequences. Mol Biol Evol. 1984;1:269–85.

## Data Availability

The datasets used and/or analysed during the current study are available from the corresponding author on reasonable request.

## Abbreviations

PCR: polymerase chain reaction
ED: emergency department
RDT: rapid diagnostic test
MSs: microsatellites
csp: circumsporozoite
DHP: dihydroartemisinin–piperaquine
AL: artemether/lumefantrine
nts: nucleotides
