# Supplementary figures and images for: A case of Plasmodium malariae recurrence: recrudescence or reinfection?
Source: Malar J. 2019 May 14;18:169. doi: 10.1186/s12936-019-2806-y (PMC6515619; doi:10.1186/s12936-019-2806-y)

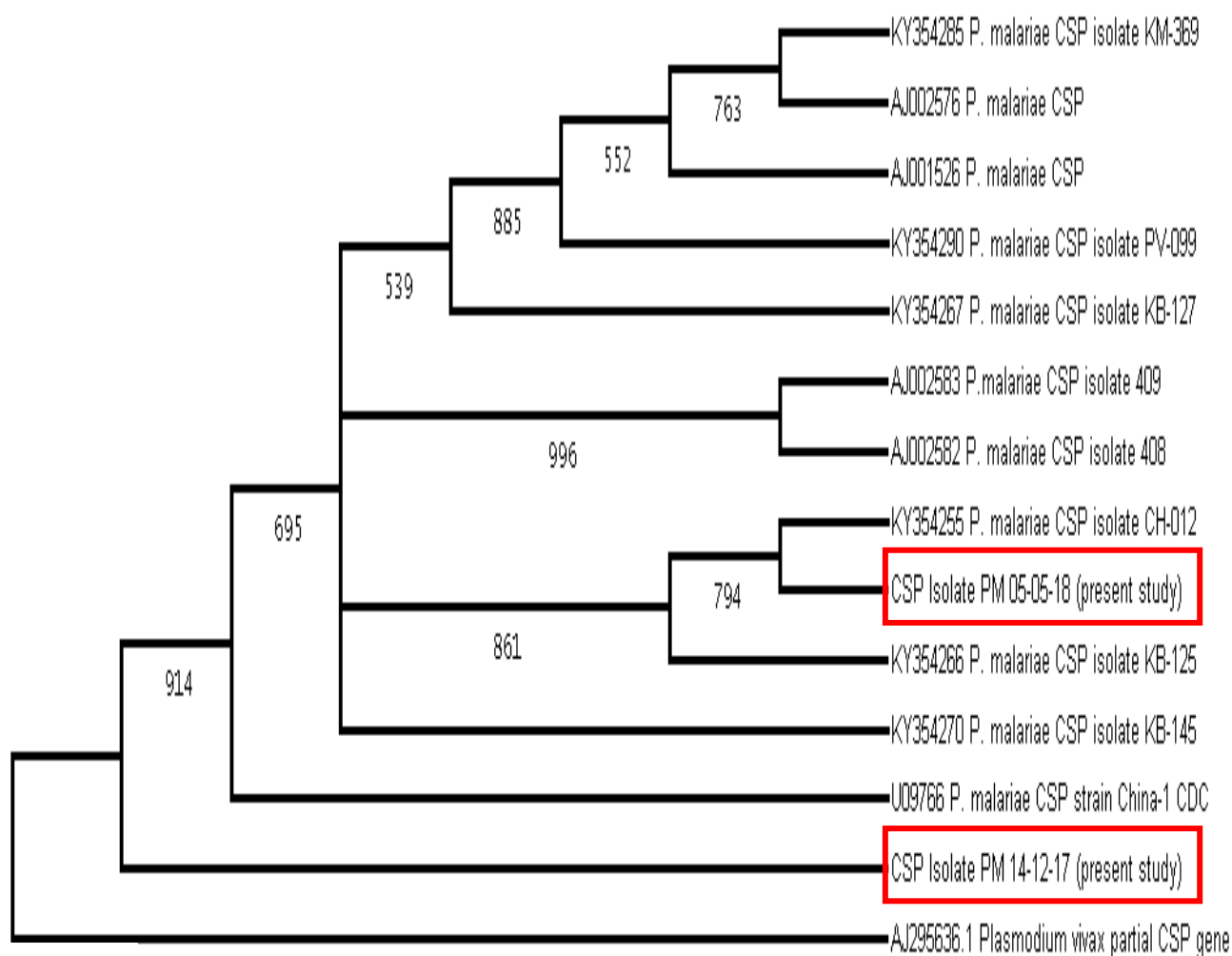

375

Supplement: Supplementary file 1 — Additional file 1: Figure S1. Phylogenetic tree inferred from CSP nucleotide sequence alignment obtained from P. malariae isolates analyzed in the present study (Isolate PM 14-12-17; Isolate PM 05-05-17) and from 12 representative CSP gene sequences retrieved from the GenBank database. P. vivax CSP gene sequence (Genbank accession number AJ295636) was also included in the analysis and used as an outgroup. Phylogenetic analysis was done using the neighbour-joining method constructed using the neighbor-joining method by bootstrapping with 1000 replicates, and phylogenetic distances were measured by Tajima-Nei model, using the Accelrys DS Gene software package (Accelrys Inc., San Diego, CA, USA). [file 12936_2019_2806_MOESM1_ESM.pdf]

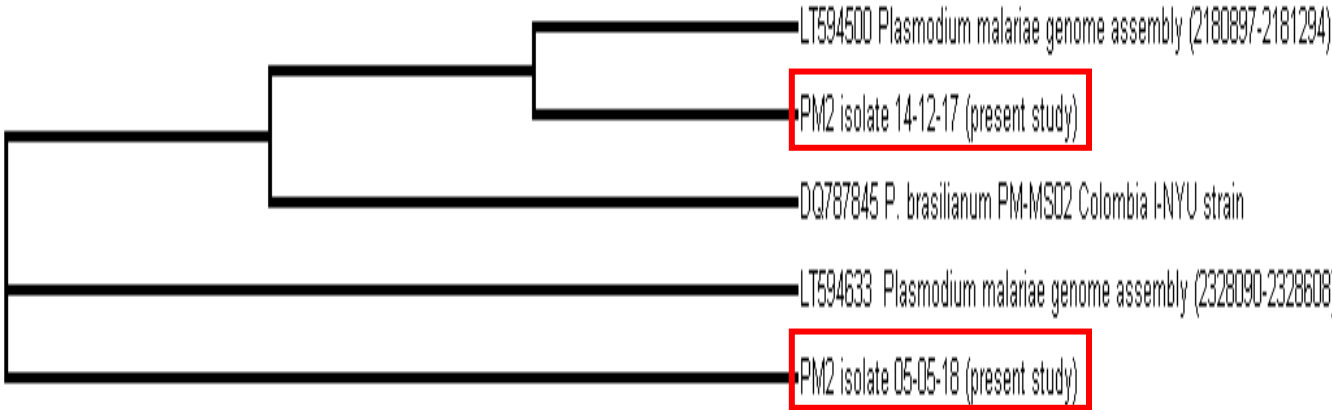

Supplement: Supplementary file 2 — Additional file 2: Figure S2. Phylogenetic tree inferred from PM2 microsatellite sequence alignment obtained from P. malariae isolates analyzed in the present study (Isolate PM 14-12-17; Isolate PM 05-05-17) and from all representative sequences of PM2 microsatellite retrieved from the GenBank database. Phylogenetic analysis was done using the neighbour-joining method constructed using the neighbor-joining method by bootstrapping with 1000 replicates, and phylogenetic distances were measured by Tajima-Nei model, using the Accelrys DS Gene software package (Accelrys Inc., San Diego,CA,USA). Reference: Tajima F, Nei M. Estimation of evolutionary distance between nucleotide sequences. Mol Biol Evol. 1984;1:269–85. [file 12936_2019_2806_MOESM2_ESM.pdf]
